# Toxicity Induced by Cytokines, Glucose, and Lipids Increase Apoptosis and Hamper Insulin Secretion in the 1.1E7 Beta Cell-Line

**DOI:** 10.3390/ijms22052559

**Published:** 2021-03-04

**Authors:** Antonia Diaz-Ganete, Aranzazu Quiroga-de-Castro, Rosa M. Mateos, Francisco Medina, Carmen Segundo, Alfonso M. Lechuga-Sancho

**Affiliations:** 1 Inflammation, Nutrition, Metabolism and Oxidative Stress Study Group (INMOX), Biomedical Research and Innovation Institute of Cádiz (INiBICA), Research Unit, Puerta del Mar University Hospital, 11009 Cádiz, Spain; tonydiazganete@gmail.com (A.D.-G.); rosa.mateos@uca.es (R.M.M.); fmedinaprieto@gmail.com (F.M.); 2 Area of Pediatrics, Department of Child and Mother Health and Radiology, Medical School, University of Cádiz, 11002 Cádiz, Spain; aranzazu.quiroga.sspa@juntadeandalucia.es; 3 Area of Biochemistry and Molecular Biology, Department of Biomedicine, Biotechnology and Public Health, University of Cádiz, 11519 Cádiz, Spain; 4 Salus Infirmorum Faculty of Nursing, Cadiz University, 11001 Cadiz, Spain; 5 Pediatric Endocrinology, Department of Pediatrics, Puerta del Mar University Hospital, 11009 Cádiz, Spain

**Keywords:** apoptosis, beta cell, cytokines, cytotoxicity, diabetes, glucotoxicity, lipotoxicity, mechanism

## Abstract

Basic research on types 1 and 2 diabetes mellitus require early stage studies using beta cells or cell lines, ideally of human origin and with preserved insulin secretion in response to glucose. The 1.1E7 cells are a hybrid cell line resulting from the electrofusion of dispersed human islets and PANC-1 cells, capable of secreting insulin in response to glucose, but their survival and function under toxic conditions remains untested. This characterization is the purpose of the present study. We treated these cells with a cytokine mix, high glucose, palmitate, and the latter two combined. Under these conditions, we measured cell viability and apoptosis (MTT, Caspase Glo and TUNEL assays, as well as caspase-8 and -9 levels by Western blotting), endoplasmic reticulum stress markers (EIF2AK3, HSPA4, EIF2a, and HSPA5) by real-time PCR, and insulin secretion with a glucose challenge. All of these stimuli (i) induce apoptosis and ER stress markers expression, (ii) reduce mRNA amounts of 2–5 components of genes involved in the insulin secretory pathway, and (iii) abrogate the insulin release capability of 1.1E7 cells in response to glucose. The most pronounced effects were observed with cytokines and with palmitate and high glucose combined. This characterization may well serve as the starting point for those choosing this cell line for future basic research on certain aspects of diabetes.

## 1. Introduction

It has been estimated that approximately half a billion people are living with diabetes mellitus (DM) worldwide, and this prevalence is projected to increase by more than 50% in the next 25 years [1], with its associated increasing rates of disability, mortality, and health care costs [2]. Classically, β-cell apoptosis and diminished insulin secretion have been central hallmarks of type 1 DM (T1DM), induced by pro-inflammatory cytokines (CKs) produced in situ by infiltrating leucocytes during insulitis [3,4]. Nevertheless, more recent evidence demonstrates that insulin secreting β-cells are also diminished by apoptotic mechanisms in type 2 DM (T2DM) induced by chronic hyperglycemia (glucotoxicity) and increased levels of free fatty acids (lipotoxicity) associated stresses [5,6]. Thus, research on the quantitative contribution of these stress stimuli to β-cell functional impairment and apoptosis is relevant to understand the DM development and support new preventive and therapeutic strategies.

In vivo pro-diabetic stress triggered by pro-inflammatory CKs, hyperglycemia, and free fatty acids have been modelled in vitro, by incubating cells with CKs, high glucose concentration, and palmitate, respectively. Correspondingly, these stress stimuli induce dysfunction and apoptosis on β-cells in primary cultures as supported by many studies in rodents but fewer in humans. Both main apoptotic pathways (the intrinsic and the extrinsic pathways), interconnected at several levels, are activated by proinflammatory CKs, high glucose, and palmitate in pancreatic β-cells [7,8,9,10,11,12]. Endoplasmic reticulum (ER) stress-induced apoptosis has also been involved in CK, free fatty acids, and high glucose toxicities to β-cells. Additionally, all these stressors have been implicated in detrimental effects on insulin secretion [10,11,12,13,14,15,16]. In the ER stress response, phosphorylation of the translation initiation factor EIF2A by EIF2AK3 is related with increased transcription of molecular chaperones such as HSPA4 and HSPA5 aiming to prevent unfolded protein accumulation.

In human cells, however, the evidence is scarce as a consequence of the growing difficulties to obtain human islets and the shortage of cell lines maintaining glucose-responsive physiology resembling that of ex vivo cells. To this end, β-cell lines of human origin constitute an invaluable tool. Two of these β-cell lines, generated by electrofusion techniques proved to release insulin in response to a glucose challenge in a stepwise physiological fashion [17]. One of these, the 1.1E7 remains to be tested regarding their sensitivity to certain diabetes-related toxic conditions in terms of apoptosis induction and insulin secretion dysfunction. Moreover, the combination of glucotoxicity and lipotoxicity (gluco-lipotoxicity), has not yet been explored in any glucose-responsive β-cell line of human origin to date. 

The aim of this study is to describe the behavior of the glucose-responsive 1.1E7 β-cell line regarding apoptosis and insulin secretion when incubated with a mix of inflammatory CKs, high glucose (Glu^h^), palmitate (Palm), and the combination of these (Palm+Glu^h^).

## 2. Results

**Figure 1:**
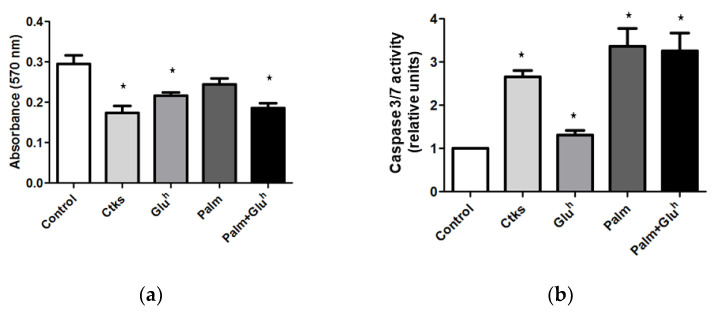

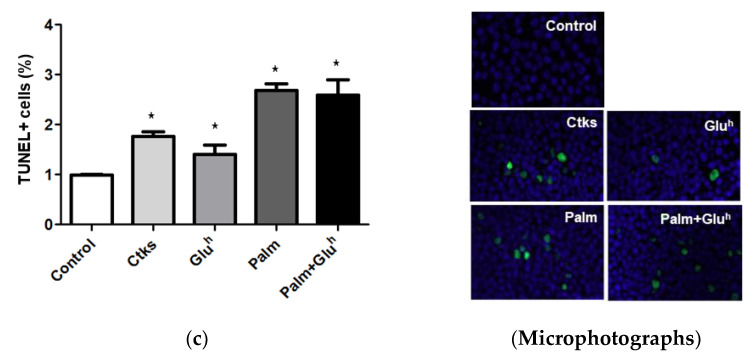
Cytokines, palmitate, high glucose, and high glucose+palmitate effect on the 1.1E7 cell survival. MTT assay for cell viability (**a**), Caspase Glo luminescence kit for caspase 3/7 activity (**b**) and TUNEL assay for apoptosis quantification (**c**) were performed to 48 h cultured 1.1E7 cells in the presence of cited stimuli. Microphotograhs are representative images of TUNEL stained cells counterstained with DAPI. Values are represented as mean ± SEM of four independent experiments. * *p* < 0.05 with to respect the control condition.

In an initial set of experiments, 1.1E7 cells were cultured in the presence of the stressor stimuli mentioned above and their metabolic viability was measured by an MTT assay. As it can be observed in Figure 1a, all stimuli except Palm decreased the 1.1E7 cells viability in a variable degree. The greatest reductions were those induced by CKs (around 50%) and Palm+Glu^h^ combination (40%), while the effect of Glu^h^ was more modest (30%).

To assess the potential role of apoptosis in this viability loss, 1.1E7 cells were treated in the conditions already described and their caspase 3/7 activation (a gold standard marker of apoptosis), was measured by a luminescence kit. As shown in Figure 1b, 1.1E7 cells treated with CKs, Palm, and Palm+Glu^h^ combination, which increased approximately 3-fold their caspase 3/7 activity with respect to the control, in contrast with the lesser effect of a single Glu^h^ stimulus. Differences between all the treatments and control and between Glu^h^ and the rest of the stimuli were significant. The presence of DNA-nicks in 1.1E7 cells (another apoptosis hallmark) was determined by TUNEL staining and microscopy. The percentage of TUNEL-positive 1.1E7 cells (Figure 1c) reproduced the pattern of caspase 3/7 activity.

**Figure 2:**
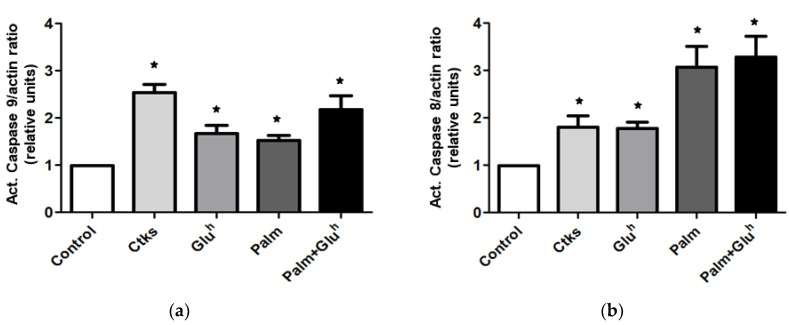
Cytokines, palmitate, high glucose, and high glucose+palmitate effect on caspase-9 and -8 activation. Active caspase-9 (**a**) and active caspase-8 (**b**) expression was measured by Western blotting in 48 h cultured 1.1E7 cells with or without the cited stimuli. The caspase/actin ratio was expressed as relative units to the control in both graphs. Values are represented as mean ± SEM of four independent experiments. * *p* < 0.05 with respect to the control condition.

In order to get a deeper insight into the apoptosis signaling involved in the 1.1E7 cells death under the assayed stimuli, the initiator caspases activation was measured by WB: Caspase-9 for the intrinsic apoptotic pathway and caspase-8 for the extrinsic one. As it can be observed in Figure 2a (representative image and mean value), all the treatments increased the amount of caspase-9 active fragment but to a different extent. The highest effect, around 2.5-fold, was obtained with the CKs exposition. Differences between all the treatments and control and between CKs and the rest of the stimuli were evidenced. Following the same experimental design, the caspase-8 activation was also explored. The results (Figure 2b) depicted a different pattern than that of caspase-9, with a modest 1.5-fold effect for CKs and Glu^h^ treatments versus a more prominent 3-fold increase for Palm and Palm+Glu^h^ mix. Differences between all the stimuli and control and between modest (CKs, Glu^h^) and prominent pattern treatments (Palm, Palm+Glu^h^) were significant.

**Figure 3:**
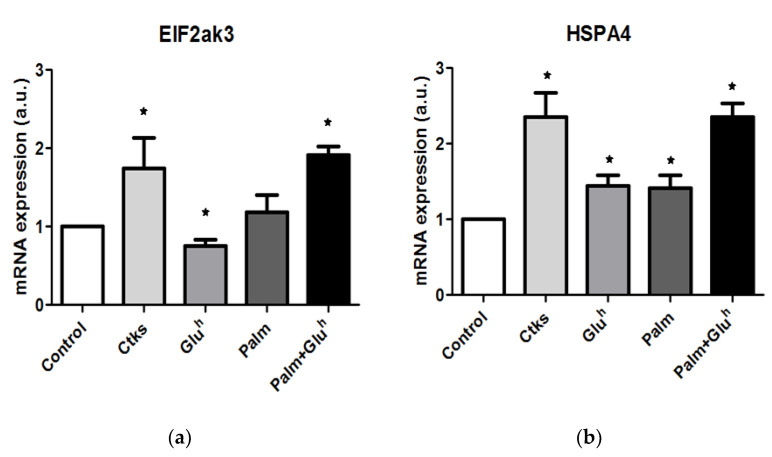

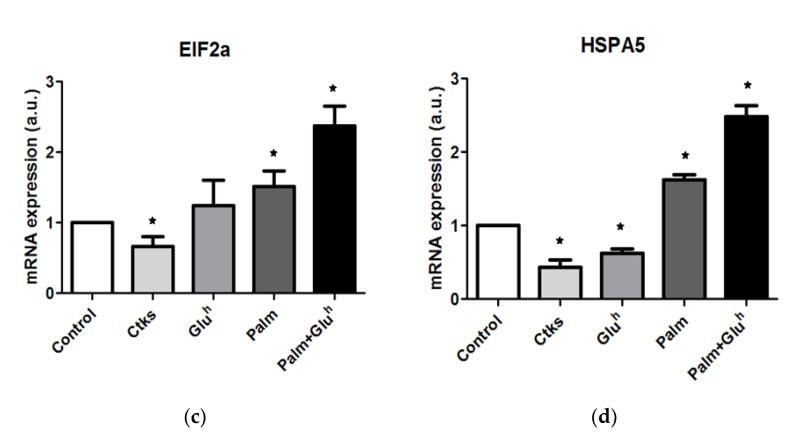
Cytokines, palmitate, high glucose, and high glucose+palmitate effect on ER stress response markers. EIF2AK3, (**a**) HSPA4, (**b**) EIF2a, (**c**) and HSPA5 (**d**) mRNA expression were measured in 48 h cultured 1.1E7 cells with or without the cited treatment. Results are presented as the mean ± SEM of the mRNA expression indicated as arbitrary units relative to the control. Values were obtained from at least four independent experiments. * *p* < 0.05 with respect to the control condition.

Considering the ER stress induction that these stimuli trigger in other β-cell systems, ER stress markers changes in 1.1E7 cells were quantified in response to our treatment panel by the real-time reverse transcription. Two of these markers, Eukaryotic translation initiation factor 2 alpha kinase 3 (EIF2ak3, Figure 3a) and Heat shock 70 kDa protein 4 (HSPA4, Figure 3b) showed a similar pattern, around a 2-fold increase for CKs and Palm+Glu^h^ treatments versus a lack of significant effect of Glu^h^ and Palm alone. The other two markers assayed Eukaryotic translation initiation factor 2A (EIF2a, Figure 3c) and Heat shock 70 kDa protein 5 (HSPA5, Figure 3d) displayed a different pattern, depicting a low or even reduced effect for CKs and Glu^h^ stimuli but a 1.5- and 2.5-fold increase with Palm and Palm+Glu^h^ treatments, respectively. Of note for all four markers, the Palm+Glu^h^ mix exerted a more pronounced effect than Glu^h^ or Palm treatment alone.

**Figure 4:**
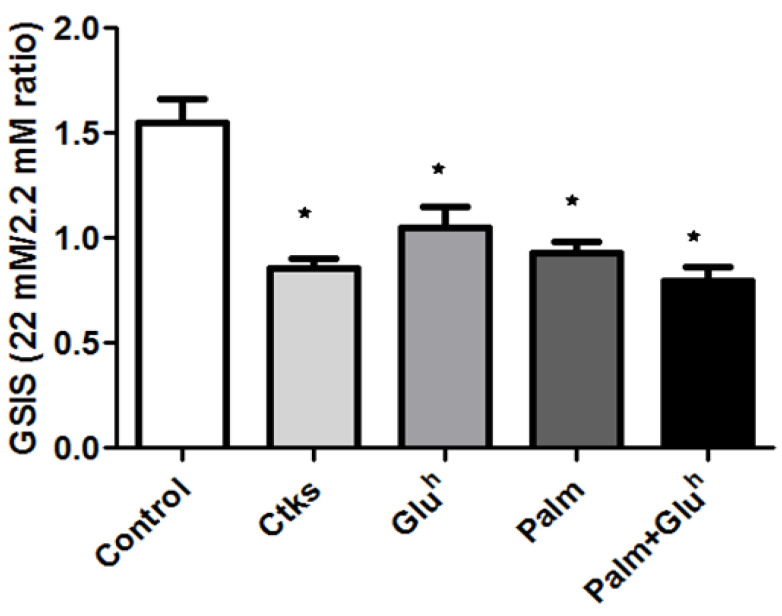
Cytokines, palmitate, high glucose, and high glucose+palmitate effect on 1.1E7 insulin secretion. Glucose stimulated insulin secretion (GSIS) was determined in 1.1E7 cells cultured during 48 h in the absence or presence of the indicated stimuli. Results are represented as the ratio of insulin secreted at the stimulatory glucose concentration (22 mM) to insulin secreted at the basal glucose concentration (2.2 mM). Values are represented as the mean ± SEM of four independent experiments. * *p* < 0.05.

The most notable feature of 1.1E7 cells, regarding basic research interests, is their insulin secretion capacity in response to glucose. To explore the effect of our stimuli in this intrinsic characteristic, 1.1E7 cells were first treated with CKs, Glu^h^, Palm or Palm+Glu^h^ and, afterwards, the glucose stimulated insulin secretion (GSIS) was quantified as the ratio of basal insulin release (as incubated with 2.2 mM of glucose for 60 min versus induced insulin release (incubating with 22 mM of glucose for 90 min). The untreated control cells responded to the glucose challenge (22 mM) increasing their basal secretion by 50%, for a mean GSIS ratio of 1.5. The remaining study conditions, CKs, Glu^h^, Palm or Palm+Glu^h^, rendered 1.1E7 unresponsive to the glucose challenge, accounting for insulin secretion equal or below the basal rate (ratio ≤1) (Figure 4).

Then, we explored if this lack of glucose induced insulin release by 1.1E7 cells could be related to an inhibitory effect of our treatment panel on the expression of genes related to their secretory function. To this end, the treated 1.1E7 cells mRNA expression of several genes was quantified by real-time reverse transcription: Insulin (*INS*), glucokinase (*GCK*), PC1/3 proprotein convertase, prohormone processing (*PCSK1*), PC2 proprotein convertase prohormone processing (*PCSK2*), K+ATP dependent channel subunit (*KCNJ11*), K_ATP_ channel subunit (*ABCC8*), Connexin 43A, Gap junction protein 1, adjacent cells communication for coordinated secretion (*GJA1*), and *PPP3CB* (Ca^2+^ signaling). The results are summarized in Table 1. None of the stimuli affected *PCSK2, ABCC8* or *PPP3CB* mRNA expression in 1.1E7 cells. The remaining genes mRNA quantities were decreased in a stimulus-dependent manner: CKs decreased all five, four of five were decreased by Glu^h^ and Palm+Glu^h^, and only two of five by the Palm treatment. *KCNJ11* mRNA, that codifies for a subunit of K+ ATP dependent channel was reduced by all four treatments. *INS* and *GJA1* mRNA were both diminished by all stimuli except Palm. The proinsulin convertase *PCSK1* mRNA amount was reduced under all treatments except Glu^h^. Finally, glucokinase (*GCK*) mRNA was only affected by CKs and Glu^h^.

## 3. Discussion

The 1.1E7 cells are being used in basic diabetes research relying on their putative close proximity to the human primary β-cell physiology [18]. Nonetheless, there is a considerable knowledge gap about the 1.1E7 cells regarding their response to stressing stimuli, broadly considered as DM inductors. This absence of evidence is especially striking in comparison with 1.1B4 (1.1E7 “sister” cell line) that accounts for more than 20 articles in this field during the last 5 years.

We intended to provide evidence on this cell line’s behavior in different well established in vitro models of diabetes. Thus, we explored the 1.1E7 cells’ response to an inflammatory environment, by treating them with a cytokine cocktail, and their behavior in conditions of high glucose (Glu^h^), palmitate (Palm), and a combination of both Palm+Glu^h.^. Of note, this approach is for an early research stage before testing on ex vivo islets, or animal models, keeping in mind that immortalized cell lines have their own peculiarities. In addition, the data obtained from them must be interpreted with caution. Regarding apoptosis, all the treatments increased both caspase 3/7 activation and TUNEL-positive 1.1E7 cells frequency, showing that Glu^h^ has a significant lower effect on death than the rest of the stimuli. Compared to 1.1B4 cells, 1.1E7 cells displayed a similar response to CKs [10] and Glu^h^ [11], but a notably lesser apoptosis induction by the Palm treatment (i.e., 2.5- fold with respect to the control for 1.1E7 cells versus 9-fold for 1.1B4 cells) [12]. Considering that apoptosis was quantified by the same technique (Caspase Glo 3/7 assay kit, Promega) and both cells lines were exposed to the same palmitate concentration (0.5 mM), the magnitude of this difference suggests that 1.1E7 cells are more resistant to palmitate lipotoxicity than 1.1B4 cells. 

The initiators caspases of the intrinsic and the extrinsic apoptotic pathways, respectively caspase-8 and -9, were appointed early as part of the apoptosis signaling network in β-cells [19]. Therefore, to gain a deeper insight of these initiator caspases’ role in 1.1E7 cells apoptosis, their activation in response to the diabetogenic stimuli was explored. Both caspase-8 and -9 were activated by all the treatments but following a different pattern: We found the highest caspase-9 activation in the cytotoxic model, while the lipotoxic and glucolipotoxic model resulted in an approximately 2-fold raise in active capase-8, suggesting that the high lipid concentration is more responsible for this effect than the high glucose concentration. To the best of our knowledge, this is the first evidence connecting the Palm treatment with the caspase-8 activation in β-cells of human origin. Of note, a recent prospective study in humans [20], revealed that the plasma caspase-8 concentration correlates with the risk of developing T2DM. Altogether, this clinical evidence and our results suggest that the plasma caspase-8, in these “prone” to T2DM subjects, might be generated by increased circulating fatty acids.

Regarding ER stress markers, 1.1E7 cells also reacted to the stimuli in different patterns: EIF2ak3 and HSPA4 increased their expression after CKs and Palm+Glu^h^ culture, while EIF2a and HSPA5 mRNA amounts increased in the presence of Palm, potentiated in combination with Glu^h^. A comparison between 1.1E7 and 1.1B4 shows a high degree of coincidence, regarding ER stress markers’ upregulation, downregulation or stability tendency in response to CKs [10], Glu^h^ [11] or Palm [12]. Although both cell lines display a similar expression pattern, 1.1B4 cells contain notably higher mRNA amounts than 1.1E7 cells in certain conditions: EIFak3 in CKs treatment (3- versus 1.8-fold with respect to the control), EIF2a in the Palm treatment (1.5- versus 2.5-fold), and HSPA5 also in the Palm treatment (1.5- versus 5.5-fold). Even a combined Palm+Glu^h^ treatment of 1.1E7 cells is able to reach only EIF2a 1.1B4 cells expression (2.5-fold), but not the HSPA5 one (2.5- versus 5.5-fold). These differences suggest that 1.1B4 cells might be more sensitive to the induced ER stress than 1.1E7 cells.

When the capability of 1.1E7 cells for glucose-driven insulin secretion was explored, a uniform reduction was obtained for all the assayed conditions. Of note, we used high glucose concentrations for the static glucose-stimulated insulin secretion assays, thus, it would be advisable to confirm these results using lower glucose concentrations (10–16.7 mmol/L), before applying them to primary cultures. In correlation with the reduced insulin secretion found, the mRNA quantities of several relevant genes for insulin processing and release were also decreased, with 4–5 components being affected for most of the stimuli, but only two of them after the Palm treatment (PCSK1 and KCNJ11). Of note, despite inhibiting only the expression of these two components, Palm was as effective as the rest of the stimuli in reducing insulin secretion by 1.1E7 cells in response to the glucose challenge. One possible explanation is that since Palm targets PCSK1 (that cleaves pro-insulin to its mature form) and KCNJ11 (a subunit of K+ ATP-dependent channel), it might be relevant enough to abrogate insulin induced secretion. Of note, the KCNJ11 expression is reduced under all the assayed stimuli, which could at least partially explain why many patients experience secondary failure to sulphonylurea therapy [21], although, again we must insist that data from cell lines must be replicated in other models before reaching solid conclusions. Anyhow, mRNA data should be confirmed by measuring protein levels. A comparison between the 1.1E7 and 1.1B4 cells secretory function shows similarities and some differences. The insulin release index in an untreated condition is lower in 1.1E7 cells than in 1.1B4 cells, 1.5 and 2.0, respectively. Nonetheless, both cells lines experience a similar decrease in their insulin induced release in response to glucose when exposed to the different toxic stimuli [10,11,12].

In summary, the toxic conditions (CKs, Glu^h^, Palm, Palm+Glu^h^) assayed on 1.1E7 cells in the present work: (i) Induce apoptosis and ER stress response, (ii) inhibit their capability to secrete insulin in response to glucose, and (iii) reduce the mRNA expression of some components of insulin secretion machinery. Of note, the presence of Palm in cultures, provided the highest apoptosis and ER stress rates of all the assayed stimuli. In addition, the prominent caspase-8 activation induced by Palm on 1.1E7 cells, an effect previously unpublished for human β-cells, might be connected with the caspase-8 recently described role as a serum early marker for T2DM in humans, though since this is data from cell lines, it must be interpreted with caution. 

## 4. Materials and Methods

### 4.1. Cell Culture

We performed the experiments on the human-derived 1.1E7 beta cell line, purchased from the Heath Protection Agency Culture Collections (Salisbury, UK, www.hpacultures.org.uk, accessed on 8 January 2021). The 1.1E7 cells were systematically cultured in a RPMI 1640 medium with the addition of 10% fetal bovine serum (FBS), 1% Penicillin-Streptomycin (Gibco, Thermo Fisher Scientific, Cheshire, UK), as well as 5.5 mM D-glucose (Merck, Darmstadt, Germany). All the incubations were performed under the following conditions: 3 × 10^5^ cells/well were cultured and grown up to 90% confluence on 6-well plaques at a 37 °C temperature and with 5% CO_2_, before setting the different toxic conditions.

Cytokines’ effects (CKs), were analyzed after incubating cultures for 48 h with a previously described cytokine cocktail containing rat IFN-γ (100 ng/ml), rat TNF-α (50 ng/ml), and recombinant human IL1-β (0.05 ng/ml) (Peprotech, Inc., London, UK), to mimic typical insulitis of preclinical diabetes at Langerhans’ Islets. Lipotoxicity (Palm) was reproduced by incubating for 48 h with 0.5 mM palmitate, obtained by dissolving sodium palmitate (Sigma, Poole, Dorset, UK), in 50% ethanol and coupled to 5% fatty acid-free bovine serum albumin (BSA) (Sigma, Poole, Dorset, UK). Glucotoxicity was approached by exposing cells to high 22 mM glucose (Glu^h^) for 48 h. Gluco-lipotoxicity (Palm+Glu^h^) was mimicked by combining Palm and Glu^h^ conditions as described above. Stimuli concentrations were set after dose-response assays.

### 4.2. MTT Assay 

Under the same incubating conditions, 30 × 10^5^ cells were cultured in 96-well plates. After 24 h, cultures were subject to the different experimental conditions: Control, CKs, Glu^h^, Palm, and Palm+Glu^h^ for 48 h. Supernatants were discarded and 25 µl of MTT solution (Merck Chemicals Ltd, Darmstandt, Germany) at a concentration of 5 mg/ml was added to each well. After a 90-min incubation at 37 °C with 5% CO_2_, the samples were washed with warm PBS. PBS was eliminated before adding 100 µl of DMSO/well. Plates were covered and gently stirred for 20 min at room temperature (RT). Then, we measured the absorbance at 570 nm.

### 4.3. Apoptosis Detection and Quantification

The apoptosis rate was analyzed performing the Dead End Fluorimetric TUNEL System (TdT-mediated dUTP Nick-End Labeling) (Promega Corp, Madison, WI, USA) following the protocol provided by the manufacturer. Briefly, an initial sample permeabilization was performed applying 0.2% Triton X-100/PBS for 20 min at RT. The samples were then washed with PBS and blocked with 4% FBS (Gibco) at RT for 30 min. The samples were then equilibrated and incubated with the nucleotide mix and the enzyme for 60 min at 37 °C. The reaction was stopped by applying 2% SCC for 15 min at RT and immediately washed with water to eliminate any fluorescein-12-dUTP excess. The samples were then cover-slipped with a mixture of DABCO (Sigma-Aldrich) (to preserve the fluorescent signal), and 0.1 mg/ml DAPI (Sigma-Aldrich) to label the nuclei. Fluorescence was visualized with a camera-coupled fluorescence microscope (Olympus BX40, Carsen Group Inc., Markham, ON, Canada). The 1000 DAPI-labeled cells were counted per sample. The results are expressed as a % of TUNEL-positive cells.

### 4.4. Caspase Assay

The activity of caspases 3/7 was determined with a Caspase Glo^®^ 3/7 assay kit (Promega, UK) following the manufacturer’s instructions. Briefly, the 1.1E7 cells were lysed in a lysis buffer. The Caspase Glo reagent was prepared immediately before use. Equal volumes of the samples and reagent were mixed and incubated for 60 min at RT, and luminescence was measured using a spectrophotometer (Biotek, EEUU). The activity was expressed as relative luminescence units.

### 4.5. Analysis of Glucose-Stimulated Insulin Secretion (GSIS)

To study how CKs, Glu^h^, Palm, and Palm+Glu^h^ affected INS1.1E7 cells’ insulin secretion in vitro, once the cells had been grown to a 90% confluence in 6-well plaques with the above described medium containing 5.5 mM D-glucose, the medium was changed to a 2.2 mM glucose for 24 h, to reduce basal insulin secretion before adding CKs, Glu^h^, Palm, and Palm+Glu^h^ to the culture media for 48 h. Then, cells were washed three times with a 2.2 mM glucose HBSS/Krebs buffer (HBBS) and incubated with the same solution for 60 min, to establish basal insulin secretion in the supernatants. Thereafter, a high glucose concentration (22 mM) HBBS was added to the cultures for 90 min. Finally, the new supernatants were collected again to measure insulin and determine GSIS.

The insulin concentration was measured using an Ultrasensitive Insulin ELISA kit (Mercodia AB, Uppsala, Sweden). Briefly, the supernatants were diluted 1:10 in a glucose-free HBSS. Then, 25 µl of each sample in duplicates were incubated for 2 h with the kit’s conjugated enzyme. After washing six times, the samples were incubated 15 min in the dark with the kit’s TMB substrate solution. Finally, the reaction was stopped by adding the corresponding solution, and the absorbance was determined at 450 nm. All the incubations were performed at RT.

### 4.6. Immunoblotting

The intrinsic and extrinsic apoptotic pathways activation in response to CKs, Glu^h^, Palm, and Palm+Glu^h^ was estimated by measuring caspase-8 and -9 levels, respectively by Western blotting. To this end, the cells were collected and centrifuged for 3 min at 3000 rpm and the supernatants were discarded. Pellets were homogenized in a pH 6.8 lysis buffer containing 125 mM Tris, 2% SDS, 1 mM DTT, Ortovanadate [1:100], and the protease inhibitor cocktail. Finally, supernatants’ protein concentration was determined by the Micro BCA kit (Thermo Fisher Scientific), according to manufacturer’s instructions.

To perform the blots, 40 μg of protein were loaded per sample, electrophoresed on 10% SDS-PAGE, and transferred to PVDF membranes (Immobilon-P, Millipore, EEUU). The membranes were then blocked for 90 min at RT with 0.1% Tween20, 5% (*w*/*v*) BSA/PBS. Then, the membranes were incubated overnight at 4 °C with the primary antibodies for caspase-9 (Medical and Biological Laboratories Co., Naka-Ku Nagoya, Japan) or caspase-8 (Cell Signaling Technology Inc, Danvers, MA, USA). Primary antibodies’ concentration was 1:1000.

Then, we washed the membranes three times in 0.1% Tween20/PBS and incubated them with the corresponding secondary antibody conjugated with peroxidase [1:2000] in 0.1% Tween20, 5% BSA/PBS, anti-Rabbit IgG (Sigma-Aldrich, EEUU) and anti-mouse IgG (Sigma-Aldrich, EEUU), during 60 min at RT. The bound peroxidase activity was visualized by the enhanced chemiluminescence kit Immun-Star WesternC (BIO-RAD, EEUU) and quantified by densitometry using the analyzer Chemidoc XRS (BIO-RAD, EEUU) and Image J software (NIH, EEUU). The results were normalized to the loading control values on each membrane. The membranes were re-probed using an antibody towards β-actin [1:2000] (Abcam), as a loading control.

### 4.7. Real-Time PCR for mRNA Quantification (qPCR)

Total RNA was isolated from our 1.1E7 cells using the NucleoSpin RNA II Kit (Macherey-Nagel), and subsequently treated with DNase. The cDNA was synthesized employing the Transcriptor First Strand cDNA Synthesis kit (Roche). The real-time quantitative PCR reaction (qPCR) was performed from cDNA using the SensiFAST SYBR No-ROX Kit (Bioline), following the manufacturer’s instructions.

As human specific primers for insulin secretory function components (*INS, GCK, PCSK1, PCSK2, KCNJ11, ABCC8, GJA1*, and *PPP3CB*) and endoplasmic reticulum (ER) stress markers (*EIF2A, EIF2AK3, HSPA4*, and *HSPA5*), we used those described in 2013 by Vasu S. et al. [11]. The forward primer sequences (5’-nt-3’) and the reverse primer sequences (5’-nt-3’) for the studied genes are listed in the table below (Table 2).

The QPCR conditions were as follows: 3 min at 95 °C initial denaturation, followed by 40 cycles of denaturating at 95 °C for 15 s, and then annealing at 60 °C for 20 s, and extension for 20 s at 72 °C. Finally, the melting curve analysis was performed at a temperature range of 60–90 °C. QPCR data were acquired with a Rotor-Gene 6000 (Corbett Research) real-time PCR detection system and subsequently analyzed applying the ΔΔCt method, with the mRNA expression normalized.

### 4.8. Statistical Analysis 

The results are expressed as the mean ± SEM of at least four independent experiments. Comparisons were tested for significance applying the Mann-Whitney statistic. Significance was considered when *p* ≤ 0.05.

## 5. Conclusions

Cytokines induce the human-derived 1.1E7 β-cell line apoptosis by activating both, intrinsic and extrinsic apoptosis pathways, as well as inducing endoplasmic reticulum stress. Additionally, they decrease these β-cells’ insulin response to glucose.

Under experimental conditions of high glucose and palmitate, apoptosis was also induced via both cascades and ER stress, and the insulin production in response to glucose was also negatively affected. Most of these effects were more pronounced when the cells were treated with a combination of the two stimuli.

Taken together, we provide evidence on this cell line’s behavior under different toxic environments, which may serve as a starting point in the early stage of basic research for certain aspects of diabetes. Notwithstanding, the data obtained from the cell lines must always be interpreted with caution.

## Figures and Tables

**Table 1:** Cytokines, palmitate, high glucose, and high glucose+palmitate effect on the expression of secretory function related genes as measured by the real-time quantitative PCR reaction. Results are presented as the mean ± SEM of mRNA expression indicated as arbitrary units.

Gene	Control	CKs	Glu^h^	Palm	Glu^h^ + Palm
*INS*	100 ± 0.7	25 ± 7 *	39 ± 4 *	77 ± 10	48 ± 11 *
*GCK*	100 ± 0.35	32 ± 8 *	42 ± 6 *	63 ± 9	65 ± 21
*PSCK1*	100 ± 0.58	6 ± 1 *	73 ± 3	39 ± 8 *	28 ± 3 *
*PSCK2*	100 ± 0.59	119 ± 7	80 ± 11	114 ± 7	103 ± 4
*KCNJ11*	100 ± 0.61	23 ± 7 *	51 ± 12 *	37 ± 10 *	33 ± 13 *
*ABCC8*	100 ± 0.58	102 ± 18	71 ± 8	136 ± 9	87 ± 17
*GJA1*	100 ± 0.46	53 ± 14 *	43 ± 13 *	82 ± 6	49 ± 5 *
*PPP3CB*	100 ± 0.58	72 ± 17	106 ± 15	129 ± 8	113 ±13

* *p* < 0.05 with respect to the control condition.

**Table 2:** List of human primers (as initially described in [11]).

Gene	Forward Primer Sequences (5’-nt-3’)	Reverse Primer Sequences (5’-nt-3’)	Amplicon Size (bp)
*INS*	F–TACCAGCATCTGCTCCCTCT	R–TGCTGGTTCAAGGGCTTTAT	120
*GCK*	F–TGGACCAAGGGCTTCAAGGCC	R–CATGTAGCAGGCATTGCAGCC	207
*PSCK1*	F–TCGCGCCTCCTAGCTCTTCGCA	R–GCAGACTCCAGGCTCTTCGCTC	173
*PSCK2*	F–TCGACCAGGTGGTGCGGGAT	R–AAAGGCGGATGTGCAGCGCT	137
*KCNJ11*	F–GAGGTAAGGAAGAGTCTGGTGGGGA	R–GCCAGGCGTGTCAGCACGTAT	224
*ABCC8*	F–ACATCTTCCGCACGCGGGTG	R–GCTGCAATGGGGATGGCCGT	115
*GJA1*	F–GCTATTGTGAATGGGGTGCT	R–CTGCCAAAATTGGGAACACT	493
*PPP3CB*	F–CAGCCCGGAAAGAAATCATA	R–ACTAGGCAACATCCCTGTGG	134
*EIF2A*	F–ACGCCGCTCTTGACAGTCCG	R–TTGCCCCAGGCAAACAAGGTCC	152
*EIF2AK3*	F–CCCCAACAAGGCCAGCCTGG	R–GGACAGCCAGCCGTGTTCCC	168
*HSPA4*	F–AGCAGCGCTCTCGGTTGCAG	R–AGACAGGACACGGACCCCCG	133
*HSPA5*	F–TGCTGCTGCCCAACTGGCTG	R–GAACACGCCGACGCAGGAGT	160

## Data Availability

All our raw data are at the laboratory private databases in an Excel format, and will be made available by the corresponding authors upon request, to anyone with scientific or publication purposes.
